# Temporal binding is enhanced in social contexts

**DOI:** 10.3758/s13423-021-01928-7

**Published:** 2021-05-04

**Authors:** David H. V. Vogel, Mathis Jording, Carolin Esser, Peter H. Weiss, Kai Vogeley

**Affiliations:** 1 Research Center Juelich, Institute of Neuroscience and Medicine, Cognitive Neuroscience (INM3), Juelich, Germany; 2 Faculty of Medicine and University Hospital Cologne, Department of Psychiatry, University of Cologne, Cologne, Germany; 3 Faculty of Medicine and University Hospital Cologne, Department of Neurology, University of Cologne, Cologne, Germany

**Keywords:** Sense of agency, Temporal binding, Time perception, Social time perception, Joint agency

## Abstract

Temporal binding (TB) refers to an underestimation of time intervals between two events, most commonly for actions and their effects. This temporal contraction is measurable for both perceived changes in social stimuli such as faces, as well as for interactions with a partner. We investigated TB in two separate experiments to uncover the individual influences of (i) participants’ belief in an interaction with a human partner (as compared to a computer), and (ii) a face-like stimulus versus an abstract stimulus mediating the interaction. The results show that TB is more pronounced when self-initiated actions result in a personal event as opposed to a mere physical effect, being suggestive of a “social hyperbinding.” The social hyperbinding effect appeared to be driven both by the belief in interacting with another person and by a face-like stimulus. However, there seemed to be no further enhancing effect when combining the top-down processes (“beliefs”) with the bottom-up processes (“perceptions”). These findings suggest a prioritization of social information for TB regardless of whether this information is introduced by top-down (beliefs) or bottom-up information (stimuli). Our results add to existing literature demonstrating an increase in action-event monitoring for social cues.

## Introduction

Jointly performed actions are assumed to enhance insight into action partners’ intentions as compared to the mere observation of others’ behaviors (Gallotti & Frith, 2013). This requires constant mutual monitoring of behavior to ensure that own actions are adequately understood and acted upon in the intended manner. This monitoring of others’ motor actions has been assumed to involve mechanisms similar to the monitoring of one´s own motor-control (Pesquita et al., 2018; Wolpert et al., 2003).

Such monitoring of actions and their effects have both been linked to the emergence of a sense of agency (SoA) (Beyer et al., 2017; Chambon et al., 2013; David et al., 2008). SoA is commonly used to refer to the experience of being in control of one’s own body, its actions, and their consequences (Gallagher, 2007; Haggard, 2017). SoA has in turn been shown to be associated with the so-called temporal binding effect (TB). TB describes the temporal contraction between a voluntary action and its consequence, hence originally referred to as “intentional binding” (Engbert et al., 2007; Haggard et al., 2002). This TB effect refers to the systematic underestimation of durations between a subject’s actions and their consequences as compared to (i) interval estimations of actions and consequences that are only observed, or (ii) interval estimations not involving action-effect relationships (for a review, see Moore & Obhi, 2012). More recent investigations have revealed that TB is not limited to action-event durations and is influenced by other factors involved in event timing, such as causation and multisensory integration (e.g., Buehner, 2012; Hoerl et al., 2020; Kirsch et al., 2019; Suzuki et al., 2019; Weller et al., 2020). Accordingly, hereafter we refer to the described effect of TB as a more adequate umbrella term instead of intentional binding.

All these factors appear to be involved in social interaction. For example, empirical studies on SoA experiences have repeatedly corroborated the specific relevance of a sense of joint agency (SoJA) for successful cooperation (Bolt et al., 2016; Dewey et al., 2014; Loehr, 2018; van der Wel, 2015), for the discrimination between “self” and “other” (David et al., 2008), for dyadic learning (van der Wel et al., 2012), and for communication based on gaze-contingent behavior (Pfeiffer et al., 2012, 2013; Recht & Grynszpan, 2019). It has been suggested that the degree of SoJA depends on the spatio-temporal predictability of the consequences (Bolt & Loehr, 2017; Brandi et al., 2019; Glover & Dixon, 2017; Pfeiffer et al., 2012; Sahaï et al., 2017; Sato, 2009; Vesper et al., 2011). Predictability also seems to be involved during the emergence of TB (Cravo et al., 2011; Ruess et al., 2017).

TB seems to be pronounced in joint actions with humans, (Obhi & Hall, 2011a; Sahaï et al., 2019), has been shown to occur for a partner’s actions just as for one’s own actions (Obhi & Hall, 2011b), and is decreased during social exclusion (Malik & Obhi, 2019). Pfister et al. (2014) demonstrated stronger TB during experienced leadership as opposed to follower situations, when estimating the durations between orders and their executions. Grynszpan et al. (2019) investigated the difference between time judgments when leading or following either a computer or another person. In the experiment two participants jointly manipulated an interconnected haptic device. Unbeknownst to the participants the device was intermittently controlled by a computer. The authors found increasingly shorter time estimates while interacting with another person and no significant TB when interacting with a computer.

Recent experimental data could demonstrate TB while leading the gaze of a face-like stimulus during joint attention states (Stephenson et al., 2018). Additionally, direct eye contact generally seems to increase TB for gaze movements (Ulloa et al., 2019). These data suggest that the presence of a social stimulus is already sufficient to result in decreases in duration judgments and hence in an increased TB.

In summary, TB provides substantial information on event processing and SoA. When put into an interpersonal context, it may provide information on processes involved in social interaction. TB appears to occur both while believing to be interacting with another person via a computer, as well as when interacting with a human-/face-like stimulus. In other words, both the belief in an ongoing human-human interaction in a top-down manner as well as the perception of a human interactant in a bottom-up fashion may cause or amplify TB.

These findings raise the question of whether TB differs between human-human interactions and human-computer interactions under the simultaneous and distinct variations of both belief and stimulus. For this purpose, we designed a TB paradigm in which we systematically compared duration estimates depending on whether the consequence of one’s own action was physical or personal, in other words, whether the action elicited a consequence in the physical world or induced a corresponding behavior of another person. We employed this paradigm in a controlled, yet believable interactive situation to investigate the change of TB between human-human interactions and human-computer interactions.

## General methods

We performed two experiments. Experiment 1 was designed to investigate whether there is any TB difference in human-human interaction and human-computer interaction under the simultaneous variation of both a cover story (i.e., belief in human-human interaction) and a stimulus. Experiment 2 was set up to disentangle the differential contributions of a cover story and stimulus. By including a confederate, participants were led to believe they were interacting with another human being. Involving a confederate has been shown to convince participants that they are really interacting with another person and thereby simulate a realistic and ecologically valid interactive social situation (Pfeiffer et al., 2014; Schilbach et al., 2010). To this end, participants were introduced to another person of the same gender and similar age as their partner for the study prior to participating in the experiment. In fact, the partner was a confederate of the experimenter and not active during the experiment. Instead, the entire experimental procedure was computer controlled.

After arriving at the test site, participants spent several minutes with their confederates for general information and informed consent, prior to being separated by a mock coin toss made out between participant and confederate. For the toss, confederates were instructed to always let the participants choose. The coin toss was rigged in favor of the participant who always won. Subsequent instructions heavily emphasized the interactive nature of the experiment by employing repeated mentions of the interaction partner and the repeated use of the words “interactive,” “together,” “cooperation.” Participants were instructed that they would act as the active part in an interactive experiment and that they would give orders to their partner via their computer by pressing either the left or the right arrow key. Thereby, the confederate would always act as the reactive partner. The confederate allegedly would be seated in front of an eye-tracker measuring their eye movements and depicting them in real time on the participants’ screen. Participants were told that the partners would be instructed to react to their orders by responding as quickly as possible by looking either to the right or the left, corresponding to the pressed arrow key, and that it was the participants’ task to estimate their partner’s reaction time.

## Experiment 1

### Methods for Experiment 1

#### Participants

We recruited 28 participants, four of whom had to be excluded after the experiment because they did not believe the cover story. Thus, 24 volunteers participated in this study (ten females, mean age 31 years (SD 10.3)). To get a vague sense of a minimum sample size we referred to the aforementioned studies on TB in social contexts (Grynszpan et al., 2019; Obhi & Hall, 2011a; Pfister et al., 2014; Sahaï et al., 2019; Stephenson et al., 2018; Ulloa et al., 2019). The paradigms used in these studies cover a broad range of designs and methods. Reported effect sizes of the social effects on TB ranged between d = 0.33 and d = 0.82. Targeting a corresponding medium effect size, we estimated a minimum sample size of 22 participants in a power analysis with a predicted effect size of Cohen’s dz = 0.55 in G*Power (Faul et al., 2007) with a desired power of 0.8. All participants reported normal or corrected-to-normal vision and hearing. Participants were included if they had no record of neurological or psychiatric disease, and if they had not been taking any neuro-psychiatric or any other psychoactive or illegal drugs for at least 2 weeks preceding the investigation.

All participants were naïve as to the purpose of the experiment. Written informed consent was given by all participants. Participants were monetarily compensated (10 €/h).

#### Stimuli and apparatus

**Fig. 1:**
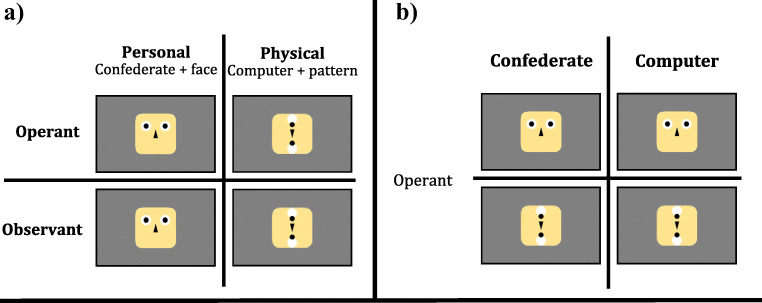
Conditions for Experiment #1 and Experiment #2: **a)** Combination of factors for *Experiment #1* are depicted on the left. Face stimulus and belief in a Confederate, as well as pattern stimulus and belief in an interaction with the computer were combined congruently. The resulting combinations (personal vs. physical) were compared across an operant and an observant condition (operant-personal, operant-physical, observant-personal, operant-physical). **b)** Combination of factors for *Experiment #2* are depicted on the right. Participants always performed key presses (operant). The initial combination between stimuli and story was separated into their respective parts, resulting in a comparison between the combined conditions of stimulus and story (confederate-face, computer-face, confederate-pattern, computer-pattern)

**Fig. 2:**
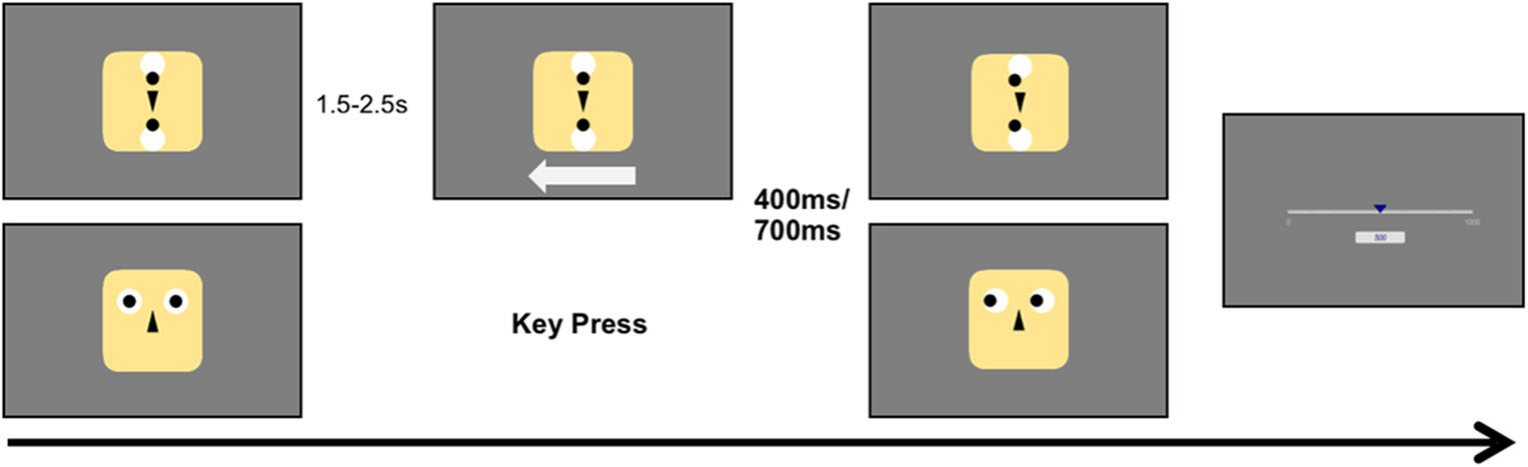
Trial event structure**.** The figure shows the set-up of Experiment 1 for the physical-observant (top row) and the personal-operant (bottom row). **Top row:** Trials started with the depiction of the respective stimulus. For observant conditions an arrow appeared after 1.5–2.5 s to indicate movement direction and to serve as the start event for the following duration judgment. After either 400 ms or 700 ms the stimulus moved its dots/eyes to the left or right depending on indicated direction. Lastly participants estimated the duration between arrow presentation and stimulus movement using a visual analog scale (VAS). **Bottom row:** Trials started with the depiction of the respective stimulus. For operant conditions participants freely pressed one of two buttons indicating a movement direction and to serve as the start event for the following duration judgment. After either 400 ms or 700 ms the stimulus moved its dots/eyes to the left or right depending on the indicated direction. Lastly participants estimated the duration between arrow presentation and stimulus movement using a VAS. During Experiment 2 participants exclusively performed the operant (key press, bottom row) conditions

We designed two different stimuli to be combined with an experimental cover story: One to represent a *person* and one to represent a *physical* object. The two types of stimuli are depicted in Figs. 1 and 2. A face stimulus was a standardized face (based on stimulus material employed in Geiger et al. (2018) constructed from simple geometric shapes). A pattern stimulus was made up of the identical geometric shapes to the face, but in a vertical and abstract arrangement not suggestive of a face. This minimalistic stimulus design allowed presentation of similar stimuli for the personal and physical conditions by arranging the same stimulus elements in two different ways. The stimuli were combined with a corresponding cover story. Whenever the face stimulus was shown, participants were additionally made to believe they were interacting with another person (henceforth *personal* partner). Whenever they were presented with the pattern stimulus, they were told to be interacting with their computer (henceforth *physical* partner).

Both stimuli were presented in three different versions during the procedure: straight, left, and right; the latter two were suggestive of changes in gaze directions in the case of faces or indicating a movement direction in the case of abstract arrangements. The experimental paradigm was programed and performed in PsychoPy2 (Peirce et al., 2019). Stimuli were presented on a 22-in. computer screen (resolution 1,680 × 1,050 pixels) against a standard grey background. Viewing distance was approximately 70 cm. A standard keyboard and mouse were used for participants’ responses.

#### Procedure

The experiment consisted of four blocked conditions of 60 trials each. Block order was counterbalanced across participants. Each block started with written and standardized oral instructions to the participants. We systematically varied three factors with two different levels, resulting in a 2 × 2 × 2 design with the factors agency (levels: *operant* vs. *observant*), partner (levels: *physical* vs. *personal*) and interval (levels: 400 vs. 700ms). Combinations of agency and partner were manipulated between blocks, while interval was varied within blocks. We chose intervals of this comparatively large duration range as earlier research by Pfeiffer et al. (2012) has indicated that intervals above a normal saccade duration of approximately 200–250 ms (Saslow, 1967; Yang et al., 2002) are necessary to create an experience of contingency during gaze interactions and that increasing durations further affect it.

We systematically varied agency by introducing *operant* and *observant* conditions (Fig. 1). In *operant* conditions, participants were instructed to press arrow keys on a keyboard to induce a movement of two black dots in the stimulus material either to the right or to the left (Fig. 1). In non-operant *observant* conditions a computer algorithm controlled the stimuli’s moving components (black circles) and participants were instructed to watch an arrow being displayed on the monitor either pointing to the right or to the left before the components moved in the indicated direction. A white arrow pointing either to the left or to the right appeared spontaneously and without participants’ involvement beneath the stimulus between 2.5 s and 3.5 s after starting a trial.

We further systematically varied the partner by presenting the moving components either as part of an arbitrary pattern arrangement or in a face-like arrangement in combination with a cover story (Fig. 1). For all *physical* conditions (both operant, observant), the pattern stimulus was presented, and participants were instructed to interact with a computer algorithm. For all *personal* conditions (both operant, observant), the face stimulus was presented, and participants were instructed to interact with a confederate.

During the *operant, personal* condition, participants were told they would be giving orders to their human partner (confederate), allegedly seated in an adjacent room. For the *observant, personal* condition participants were told they would be watching as their human partners responded to stimuli given to them by the computer.

For all *physical* conditions, instructions were the same as for *personal* conditions with the difference that during *operant, physical* conditions they would be giving orders to the computer and during physical-observant conditions they would be watching two stimuli presented by the computer.

To improve the credibility of the cover story, before starting each *personal* condition, a notification reading “Connecting to Partner Computer…” was presented on the participants’ screen before starting each *personal* block. The notification was paired with a scripted mock phone call to the pretend second test room. In addition, we introduced a 1/6 error rate over all conditions to increase credibility (fail trials). Participants were instructed that errors during interactions with the confederate were to be expected and that an artificial error rate during conditions without the interaction partner was necessary for reasons of statistical analysis.

For the factor interval, we introduced two different fixed latencies. Dots moved after either 400 ms or 700 ms (randomized across trials) following the participants’ key press (*operant*) or the algorithm-based arrow (*observant*). In all conditions, 1.5–2.5 s after each trial, participants were instructed to estimate the duration of the interval using an analog scale ranging from 0 to 1,000 ms using their computer mouse. Trials were presented in four blocks under systematic variation of the factors agency and partner counterbalanced across participants. The different durations were randomized within blocks.

This design resulted in the four blocks of the experiment being made up of *operant-personal*, *operant-physical*, *observant-personal*, and *observant-physical* conditions.

After the experiment, but prior to revealing the deception, participants underwent a structured interview with the questions: “Did you feel in control during the interaction with the other person?”; “Did you feel in control during the interaction with the computer?”; “Did anything seem off to you during the experiment?”; “Despite being so similar to the computer interaction, did the interaction with your partner seem like a real human-human interaction to you?” Afterwards, participants were fully debriefed, and the cover story was revealed. Participants who stated they had seen through the cover story either during the interview and/or during the debriefing were excluded from data analysis.

Data analysis was conducted using SPSS 25 (IBM Corp., 2017) and the R-based (R core team, 2018) software jamovi (The jamovi Project, 2019).

Post-experimental interview questions were screened and analyzed using a deductive analytical method (Mayring, 2015). Answers indicating agreement with realness of the interaction, control over the computer, or control over the interaction partner received a score of 1, whereas answers indicating the contrary received a score of 0. We calculated on a group level a realness score, a *personal* control score, and a *physical* control score by taking the mean of each answer category across the group. Scores of 1 indicate full group agreement; scores of 0 indicate no agreement.

### Results for Experiment 1

**Fig. 3:**
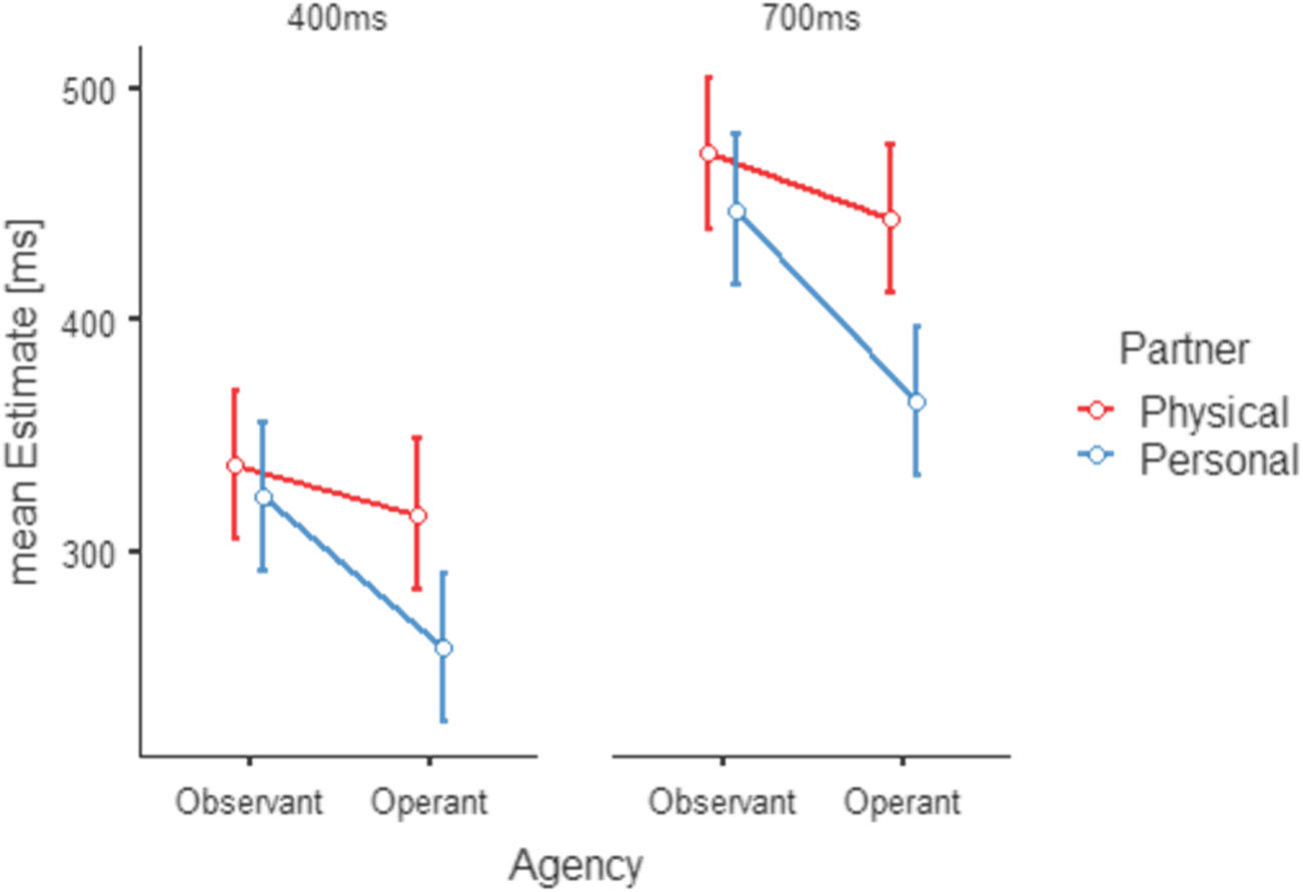
Results for Experiment 1. Mean time estimates (separately depicted for 400-ms delays in the left graph and 700-ms delays in the right graph) for the physical stimuli (red) and the personal stimuli (blue). The temporal binding effect is illustrated by the difference between observant and operant conditions. The binding effect is stronger for personal conditions as compared to physical conditions. Standard error bars area adjusted according to O’Brien and Cousineau (2014)

We hypothesized that the introduction of a simultaneous alteration in both cover story and stimulus would elicit significantly smaller duration estimates for human-human interaction latencies than for human-computer interaction latencies corresponding to a larger TB. Figure 3 illustrates the key results from a 2 × 2 × 2 repeated-measures analysis of variance (agency × partner × interval) on the participants’ mean duration estimates excluding fail trials. There was a main effect for INTERVAL (*F*(1,23) = 36.922, *p* < .001, ƞ^2^ = 0.616), indicating that participants correctly differentiated between the two delay intervals of 400 ms and 700 ms. Participants underestimated the duration of the intervals more strongly during operant conditions (main effect for agency; *F*(1,23) = 11.787, *p* = 0.002, ƞ^2^ = 0.339) and for the personal interaction (main effect of partner; *F*(1,23) = 6.513, *p* = 0.018, ƞ^2^ = 0.221). However, the strong interaction between partner and agency (*F*(1,23) = 11.019, *p* = .003, ƞ^2^ = 0.324), indicating that the temporal binding effect was stronger during interactions with a person as compared to interactions with physical objects, constitutes the main finding. This stronger TB in the socially interactive condition conversely suggests a stronger implicit SoA. No significant interactional effect with the factor interval could be found.

Post-experimental interview question analysis yielded group scores for realness of 0.92 for the personal condition, indicating strong belief in the human-human interaction. The score for *physical* control was 0.75. The score for *person* control was 0.96.

### Discussion for Experiment 1

Experiment 1 aimed at investigating TB during a person-oriented interactive situation as compared to an object-oriented non-interactive situation. Participants judged durations to be longer for longer time intervals. This finding validates participants’ ability to generally judge time intervals. Although, on a group level, both durations were underestimated, the differentiation of intervals was sufficiently performed.

With respect to the factors agency and partner, time estimates were significantly lower during operant as compared to observant conditions, independent of whether participants thought to interact with another person as opposed to an object. Participants systematically underestimated durations when they were acting to cause an event as compared to watching two causally linked events without performing a button press. This finding corresponds to the so-called intentional binding effect as the temporal binding of an action and its consequence (Engbert et al., 2007; Haggard et al., 2002; Moore & Obhi, 2012).

Importantly, for the socially enhanced human-human interactions, interval judgments were significantly lower than for human-computer situations. Depending on whether participants were watching or interacting with a person or an object, time intervals were judged lower, suggesting a pronounced TB for human-human interactions. This finding confirms our first hypothesis of stronger TB for social events and extends the initially described findings on TB when performing actions relating to a human partner (Grynszpan et al., 2019; Obhi & Hall, 2011b; Pfister et al., 2014) or to a face-like stimulus (Stephenson et al., 2018; Ulloa et al., 2019).

## Experiment 2

### Methods for Experiment 2

#### Participants

We recruited 36 participants. Four participants had to be excluded because they did not believe the cover story. Thus, 32 participants were included in the experiment (17 female, mean age 28.7 years (SD 11.2)). A sample size of a minimum of 31 participants was determined by a power analysis of the effect sizes for the interaction effect between partner and agency found in Experiment 1 using Cohen’s dz = 0.68 in G*Power (Faul et al., 2007) with a desired power of 0.95. All participants reported normal or corrected-to-normal vision and hearing. Participants were included if they had no record of neurological or psychiatric disease, and if they had not been taking any neuro-psychiatric or any other psychoactive or illegal drugs for at least 2 weeks preceding the investigation. All participants were naïve as to the purpose of the experiment. Written informed consent was given by all participants. Participants were monetarily compensated (10 €/h).

#### Stimuli and apparatus

The stimuli used in Experiment 2 were identical to those used in Experiment 1 (see Fig. 1). Stimulus presentation and the apparatus used were identical to Experiment 1.

#### Procedure

Experiment 2 was designed as a variation of Experiment 1 intended to differentiate between the influence of the *pattern* versus the *face* stimulus and the influence of the cover story, i.e., interacting either with a *computer* or with a *confederate* (Fig. 1). To this end, we used the design of the driving effect of Experiment 1, namely the stronger underestimation during social interactions. To this end, we dropped the observant conditions from Experiment #1 and participants always performed key presses. Essentially, the factor agency was no longer part of the design and participants now always gave orders to either their confederate, or to the computer.

We divided the factor partner from Experiment 1 into the two factors story and stimulus. Story was made up of *confederate* and *computer* and reflected the cover story relating to the respective part of the experiment*.* Stimulus entailed the *face* and the *pattern* stimuli used in Experiment 1. Interval again contained durations of 400 ms and 700 ms, just as in Experiment 1.

Accordingly, Experiment 2 consisted of a 2 × 2 × 2 factorial design with the factors story (levels: *computer* vs. *confederate*), stimulus (levels: *face* vs. *pattern*), and interval (levels: 400 vs. 700 ms). Story and stimulus were presented block-wise, while interval was randomized within blocks.

After having been separated from their alleged interaction partners, participants were instructed similarly to Experiment 1 that they were to perform the parts of the experiment either with their partners (*confederate* condition) or with the computer (*computer* condition). For the two *confederate* blocks and the two *computer* blocks, participants were instructed that they were to be shown either the *face* or the *pattern* stimulus. Unlike Experiment 1, stimulus presentation afforded no indication as to the nature of the story, just as the type of story did not predict the stimulus. This design resulted in the four blocks of the experiment being made up of *confederate-face*, *confederate-pattern*, *computer-face*, and *computer-pattern* conditions. The two conditions *confederate-face* and *computer-pattern* were identical to the *operant* conditions of Experiment 1.

Data analysis was conducted using SPSS 25 (IBM Corp., 2017) and the R-based (R core team, 2018) software jamovi (The jamovi Project, 2019).

### Results for Experiment 2

**Fig. 4:**
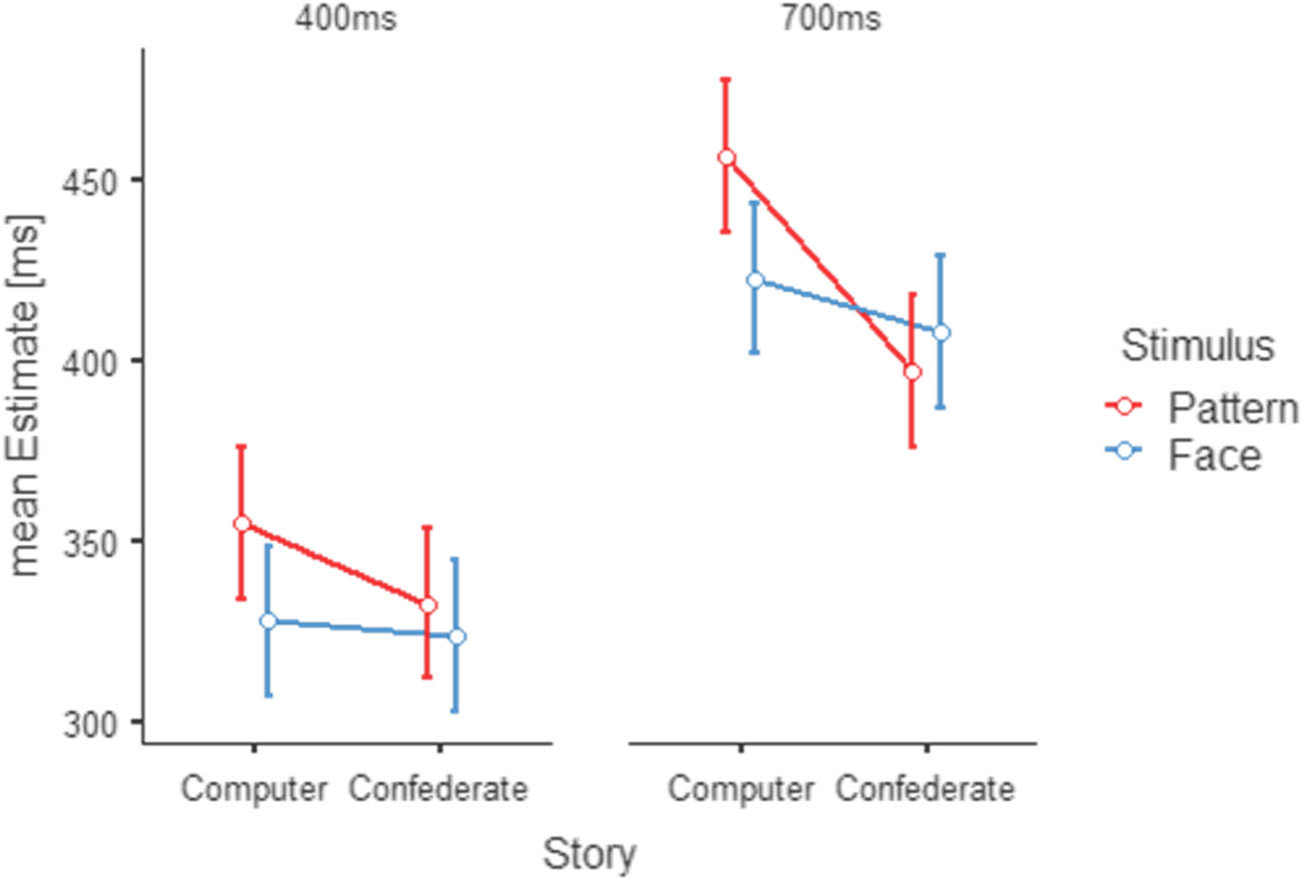
Results for Experiment 2. Mean time estimates (separately depicted for 400-ms delays in the left graph and 700-ms delays in the right graph) for the pattern stimuli (red) and the face stimuli (blue). The belief in a human-computer vs. a human-human interaction is depicted separately within graphs. Temporal binding between conditions was detectable during interactions when the stimulus depicted a face (blue). TB was stronger for belief in a human interactant. This effect of belief was not further enhanced by changes in stimulus appearance. Standard error bars are adjusted according to O’Brien and Cousineau (2014)

We hypothesized that both the factor story and the factor stimulus would significantly shorten time estimates. We further predicted that both factors would significantly interact with each other to further decrease time estimates. Figure 4 illustrates the results for Experiment 2. We calculated a 2 × 2 × 2 repeated-measures analysis of variance (story × stimulus × interval) on the participants’ mean duration estimates excluding fail trials. We found main effects for story (*F*(1,31) = 4.85, *p* = 0.035, ƞ^2^ = 0.135) and for interval (*F*(1,31) = 48.95, *p* < .001, ƞ^2^ = 0.612). We found two significant two-way interactions, namely between story and stimulus (F(1,31) = 5.85, p = 0.022, ƞ^2^ = 0.159) and story and interval (F(1,31) = 16.09, p < 0.001, ƞ^2^ = 0.342). Lastly, the three-way interaction between story, stimulus, and interval reached statistical significance (*F*(1,31) = 7.17, *p* = 0.012, ƞ^2^ = 0.188). No other interactions reached statistical significance.

For the interaction story by interval, post hoc comparison by paired-sample t-tests using Bonferroni corrections for multiple comparisons revealed that the interaction effect was primarily driven by a significant difference between the estimates for the 700-ms intervals (*computer* vs. *confederate; p* = 0.002), while estimates were similar for the 400-ms intervals (p > 0.999).

As in Experiment 1, post-experimental interview questions were screened and analyzed using a deductive analytical method (Mayring, 2015). Group scores for realness were 0.81. The score for *physical* control was 0.71. The score for *person* control was 0.81.

### Discussion for Experiment 2

Experiment 2 was designed to differentially analyze the individual and interactional effects of the two manipulated interactional qualities as variation of a top-down process (story) and a bottom-up process (stimulus). We were able to confirm our hypothesis concerning the factor story in Experiment 1, showing that duration estimates were significantly shorter for durations involving an interaction with a confederate.

Our data show an influence of stimulus appearance during interactions with the computer. Durations were judged to be shorter when a face was displayed. This effect of the face seems similar to the effect of the belief in a confederate but does not seem to enhance the story’s effect any further when both personal variations (confederate and face) are combined.

We found that at the longer duration of 700 ms these effects appeared to be stronger than at 400 ms as demonstrated by the significant interactions between story and interval, as well as the significant three-way interaction. We interpret this influence of the duration to be caused by a floor effect of the relative underestimation underlying TB. As the shorter duration of 400 ms is comparatively close to human reaction time, there is not much room for further underestimation of this duration by TB. In contrast, the differential underestimation when interacting with a confederate becomes clearly visible for the longer duration of 700 ms. The same holds true for the interaction effect between story and stimulus, which was more pronounced for the larger durations.

These findings can be interpreted as relative decreases in duration estimates whenever the overall impression of the interaction partner appeared in any way human – either by story or by stimulus. However, belief in humanness and human appearance did not cumulate, indicating that any sufficient information about humanness might be enough to increase TB.

## General discussion

The two experiments reported herein were designed to investigate the influence of successful and cooperative interpersonal interaction on time estimation. To this end, we investigated: (i) TB using a combination of a cover story involving a confederate and a face-like stimulus material in passive (*observant*) and active conditions (*operant*) in Experiment 1, and (ii) TB during active conditions with a systematic variation of the cover story with the same stimulus material in Experiment 2. While Experiment 1 led to a comparatively clear picture of “social hyperbinding” essentially reproducing earlier findings on TB in interactive situations (Grynszpan et al., 2019; Obhi & Hall, 2011b; Pfister et al., 2014) and social stimuli (Stephenson et al., 2018; Ulloa et al., 2019), the results from Experiment 2 informed about the differential roles of the top-down processes and the bottom-up processes involved.

Results from Experiment 1 show that the observed increase in underestimation of time intervals for interactions is mediated by the combination of an assumed partner and their visualization as a face-like stimulus. Earlier studies have demonstrated similar effects for both human-human interactions (Grynszpan et al., 2019; Obhi & Hall, 2011b; Pfister et al., 2014) and actions directed at face-like stimuli (Stephenson et al., 2018; Ulloa et al., 2019). In summary, Experiment 1 indicates that a combination of a belief in a human-human interaction with a face-like stimulus elicits an increased TB. As Experiment 2 suggests, this effect does not appear to be different from TB triggered by the exposition to a face without belief or by a belief in a human-human interaction without a face-like stimulus.

As compared to earlier investigations using face-like stimuli, the component of interacting with a confederate constitutes the most substantial and informative difference of our study. Our results suggest that TB is reliably observed when both influential factors (belief and stimulus appearance) are introduced simultaneously (Experiment 1). Experiment 2 suggests that stimulus per se does not have an added effect over and above the top-down influence of the cover story. Hence, the results of the two experiments reinforce the assumption that the perceived humanness of the interaction partner influences time perception substantially and reflects the socially interactive situation.

The information necessary for TB to emerge can be elicited by the belief to interact with a human counterpart or by the percept depicting a human. Irrespective of whether the stimulus appeared as a face or participants believed their partner was human, the interaction was experienced as socially contextualized. Conversely, only in the condition in which sources of both social and personal information were absent, this context was not established. Yet, either source alone was sufficient. Once the situation was established as a social interaction any additional personal information did not modulate the experience any further.

Differences in duration judgments of assumed human partners might have been influenced by prior assumptions on usual reaction times, or by social desirability to judge humans to be faster than computers. While this might be true for the results from Experiment 1, results from Experiment 2 suggest that other mechanisms should be considered. As TB was also measurable for computer conditions with a face-like stimulus, it is unlikely that the similar effect for human-human interactions should be solely caused by confounding beliefs. Instead, we propose that TB rather depends on the overall belief in a social action partner, which in turn substantially changes the perception of the respective stimulus.

An important determinant of TB is the predictability of the event elicited by prior action (Cravo et al., 2011; Ruess et al., 2017). Our findings may be explained by a higher predictability of actions by other persons, as compared to those by physical objects. At first glance, reactions from objects may be more predictable than those by other people, as they purely rely on the influence of external physical forces (e.g., Heider, 1958). However, predictability also relies on prior information (Teufel & Fletcher, 2020). Additional information about other persons and their potential behavior not available in objects, such as, for example, gaze information or information from mentalizing, has been shown to increase the cognitive processing speed during social encounters (Itier et al., 2006; Rousselet et al., 2008). By this mechanism, the contingency between action and outcome is increased, and the attribution of causality in the social context is even more pronounced (for a recent discussion, see Fereday et al., 2019). More generally, such an increased monitoring for social cues necessary to process additional information might also withdraw attentional resources from time perception processes, resulting in smaller duration judgments during social action (Polti et al., 2018; Zakay, 2014).

Such a proposed mechanism is in line with those assumed to underlie TB. A mechanism relying on causation attributions and a subsequently increased monitoring of events following a given action (e.g., Buehner, 2012; Hoerl et al., 2020) could indeed explain our results. In our experimental context, the correct belief about the causal consequence of an event (or action) results in smaller duration estimates and hence TB. Arguably, actions relating to a face-like stimulus or to an assumed human being might trigger more specific assumptions about the stimuli’s behavior than when directing action towards a geometric figure. In other words, we might have specific assumptions on how faces will react to our actions, irrespective of what we know about the nature of the agent behind the face, as well as on how other persons will react to our actions, irrespective of what they look like.

Similarly, a multisensory or cue integration approach may explain the observed TB (e.g., Kirsch et al., 2019; Weller et al., 2020). The smaller duration judgments are thus explained by an increase in the monitoring of relevant perceptual information when an action and a subsequent signal are perceived as part of a single event. With respect to the current study, situations including relevant social information are more likely to be perceived as connected events, or sensory information is monitored more closely due to its socially induced relevance. Again, this increased monitoring is determined not exclusively by visual information about the stimulus, but also by prior beliefs about the situation.

Such an explanation could be further supported by the assumption of mentalizing as a key process in social interactions. The ascription of a specific internal state or state of mind to an interaction partner during an ongoing social encounter is what makes the interaction with persons inherently different from actions performed on objects. For both gaze perception and mentalizing processes, similar neural mechanisms have been suggested to be involved (Carlin & Calder, 2013; Nummenmaa et al., 2010; Vogeley, 2017). Our findings may therefore inspire further research into the neural aspects of increased binding during social interaction.

Taken together, the presented findings contribute to the recent hypothesis of socio-motor action control, which suggests a substantial influence of socio-cognitive processes on sensorimotor mechanisms (Kunde et al., 2017). It proposes that during interaction with another person, actions are selected based on their most likely social consequences. Action-effect monitoring and hence their respective predictions are boosted by social cues. Importantly, this implies that social interactions are influenced by both bottom-up and top-down mechanisms, and exactly this is suggested by our results. As stated above, such privileged monitoring and better predictability may foster successful interaction with a partner (Bolt & Loehr, 2017; Brandi et al., 2019; Glover & Dixon, 2017; Pfeiffer et al., 2012; Sahaï et al., 2017; Sato, 2009; Vesper et al., 2011), and might serve to combat the variability of human behavior (Pfister et al., 2020).

## Conclusions

This study shows that compared to self-initiated physical action, TB in self-initiated social action, better referred to as inter-action, is substantially more pronounced in the sense of a “social hyperbinding.” The effect appears to be similarly driven by the belief of interacting with another person as a top-down influence as well as by the bottom-up influence of the stimulus material. However, neither source of social information seems to have any added influence on TB. This finding provides further evidence for the fundamental conceptual difference between persons and things (Heider, 1958; Vogeley, 2017). The results suggest that during interactions involving social cues, action-effect monitoring is increased and influences time perception. This increased monitoring may underlie successful interaction and the emergence of a SoJA.

## References

[CR1] Beyer F, Sidarus N, Bonicalzi S, Haggard P (2017). Beyond self-serving bias: diffusion of responsibility reduces sense of agency and outcome monitoring. Social Cognitive and Affective Neuroscience.

[CR2] Bolt NK, Loehr JD (2017). The predictability of a partner’s actions modulates the sense of joint agency. Cognition.

[CR3] Bolt NK, Poncelet EM, Schultz BG, Loehr JD (2016). Mutual coordination strengthens the sense of joint agency in cooperative joint action. Consciousness and Cognition.

[CR4] Brandi ML, Kaifel D, Bolis D, Schilbach L (2019). The Interactive Self–A Review on Simulating Social Interactions to Understand the Mechanisms of Social Agency. i-com.

[CR5] Buehner MJ (2012). Understanding the past, predicting the future: causation, not intentional action, is the root of temporal binding. Psychological science.

[CR6] Carlin JD, Calder AJ (2013). The neural basis of eye gaze processing. Current opinion in neurobiology.

[CR7] Chambon V, Wenke D, Fleming SM, Prinz W, Haggard P (2013). An online neural substrate for sense of agency. Cerebral Cortex.

[CR8] Cravo AM, Claessens PM, Baldo MV (2011). The relation between action, predictability and temporal contiguity in temporal binding. Acta Psychologica.

[CR9] David N, Newen A, Vogeley K (2008). The “sense of agency” and its underlying cognitive and neural mechanisms. Consciousness and cognition.

[CR10] Dewey JA, Pacherie E, Knoblich G (2014). The phenomenology of controlling a moving object with another person. Cognition.

[CR11] Engbert K, Wohlschläger A, Thomas R, Haggard P (2007). Agency, subjective time, and other minds. Journal of Experimental Psychology: Human Perception and Performance.

[CR12] Faul F, Erdfelder E, Lang AG, Buchner A (2007). G* Power 3: A flexible statistical power analysis program for the social, behavioral, and biomedical sciences. Behavior Research Methods.

[CR13] Fereday R, Buehner MJ, Rushton SK (2019). The role of time perception in temporal binding: Impaired temporal resolution in causal sequences. Cognition.

[CR14] Gallagher S (2007). The natural philosophy of agency. Philosophy Compass.

[CR15] Gallotti M, Frith CD (2013). Social cognition in the we-mode. Trends Cogn Sci.

[CR16] Geiger A, Cleeremans A, Bente G, Vogeley K (2018). Social cues alter implicit motor learning in a serial reaction time task. Frontiers in human neuroscience.

[CR17] Glover S, Dixon P (2017). The role of predictability in cooperative and competitive joint action. Journal of Experimental Psychology: Human Perception and Performance.

[CR18] Grynszpan O, Sahaï A, Hamidi N, Pacherie E, Berberian B, Roche L, Saint-Bauzel L (2019). The sense of agency in human-human vs human-robot joint action. Consciousness and Cognition.

[CR19] Haggard P, Clark S, Kalogeras J (2002). Voluntary action and conscious awareness. Nature neuroscience.

[CR20] Haggard P (2017). Sense of agency in the human brain. Nature Reviews Neuroscience.

[CR21] Heider F. (1958) *The psychology of interpersonal relations*. Jon Wiley and Sons.

[CR22] Hoerl C, Lorimer S, McCormack T, Lagnado DA, Blakey E, Tecwyn EC, Buehner MJ (2020). Temporal binding, causation, and agency: Developing a new theoretical framework. Cognitive Science.

[CR23] IBM Corp. (2017). *IBM SPSS Statistics for Windows*, Version 25.0. IBM Corp.

[CR24] Itier RJ, Latinus M, Taylor MJ (2006). Face, eye and object early processing: what is the face specificity?. Neuroimage.

[CR25] The jamovi project (2019). *jamovi. (Version 1.1)* [Computer Software]. Retrieved from https://www.jamovi.org.

[CR26] Kirsch W, Kunde W, Herbort O (2019). Intentional binding is unrelated to action intention. Journal of Experimental Psychology: Human Perception and Performance.

[CR27] Kunde, W., Weller, L., & Pfister, R. (2017). Sociomotor action control. *Psychonomic Bulletin & Review*, 1–15. 10.3758/s13423-017-1316-6 28560533

[CR28] Loehr JD (2018). Shared credit for shared success: Successful joint performance strengthens the sense of joint agency. Consciousness and Cognition.

[CR29] Malik RA, Obhi SS (2019). Social exclusion reduces the sense of agency: Evidence from intentional binding. Consciousness and Cognition.

[CR30] Mayring, P. (2015). Qualitative content analysis: Theoretical background and procedures. In *Approaches to qualitative research in mathematics education* (pp. 365–380). Springer, Dordrecht.

[CR31] Moore JW, Obhi SS (2012). Intentional binding and the sense of agency: a review. Consciousness and Cognition.

[CR32] Nummenmaa L, Passamonti L, Rowe J, Engell AD, Calder AJ (2010). Connectivity analysis reveals a cortical network for eye gaze perception. Cerebral Cortex.

[CR33] Obhi, S. S., & Hall, P. (2011a). Sense of agency in joint action: Influence of human and computer co-actors. *Experimental Brain Research*, 211(3–4), 663–670. 10.1007/s00221-011-2662-7 21503652

[CR34] Obhi, S. S., & Hall, P. (2011b). Sense of agency and intentional binding in joint action. *Experimental Brain Research*, 211(3–4), 655. 10.1007/s00221-011-2675-2 21503647

[CR35] O’Brien F, Cousineau D (2014). Representing error bars in within-subject designs in typical software packages. The Quantitative Methods for Psyschology.

[CR36] Pesquita A, Whitwell RL, Enns JT (2018). Predictive joint-action model: A hierarchical predictive approach to human cooperation. Psychonomic Bulletin & Review.

[CR37] Peirce, J. W., Gray, J. R., Simpson, S., MacAskill, M. R., Höchenberger, R., Sogo, H., Kastman, E., Lindeløv, J. (2019). PsychoPy2: experiments in behavior made easy. *Behavior Research Methods*. 10.3758/s13428-018-01193-y 10.3758/s13428-018-01193-y PMC6420413 30734206

[CR38] Pfeiffer U, Schilbach L, Timmermans B, Jording M, Bente G, Vogeley K (2012). Eyes on the mind: investigating the influence of gaze dynamics on the perception of others in real-time social interaction. Frontiers in Psychology.

[CR39] Pfeiffer UJ, Vogeley K, Schilbach L (2013). From gaze cueing to dual eye-tracking: novel approaches to investigate the neural correlates of gaze in social interaction. Neuroscience & Biobehavioral Reviews.

[CR40] Pfeiffer UJ, Schilbach L, Timmermans B, Kuzmanovic B, Georgescu AL, Bente G, Vogeley K (2014). Why we interact: on the functional role of the striatum in the subjective experience of social interaction. NeuroImage.

[CR41] Pfister R, Obhi SS, Rieger M, Wenke D (2014). Action and perception in social contexts: intentional binding for social action effects. Frontiers in human neuroscience.

[CR42] Pfister R, Weller L, Kunde W (2020). When actions go awry: Monitoring partner errors and machine malfunctions. Journal of Experimental Psychology: General.

[CR43] Polti I, Martin B, van Wassenhove V (2018). The effect of attention and working memory on the estimation of elapsed time. Scientific Reports.

[CR44] Recht, S., & Grynszpan, O. (2019). The sense of social agency in gaze leading. *Journal on Multimodal User Interfaces*, 1–12.

[CR45] Ruess M, Thomaschke R, Kiesel A (2017). The time course of intentional binding. Attention, Perception, & Psychophysics.

[CR46] R Core Team (2018). *R: A Language and envionment for statistical computing*. [Computer software]. Retrieved from https://crasn.r-project.org/.

[CR47] Rousselet GA, Husk JS, Bennett PJ, Sekuler AB (2008). Time course and robustness of ERP object and face differences. Journal of Vision.

[CR48] Sahaï A, Desantis A, Grynszpan O, Pacherie E, Berberian B (2019). Action co-representation and the sense of agency during a joint Simon task: Comparing human and machine co-agents. Consciousness and Cognition.

[CR49] Sahaï A, Pacherie E, Grynszpan O, Berberian B (2017). Predictive mechanisms are not involved the same way during human-human vs. Human-machine interactions: a review. Frontiers in Neurorobotics.

[CR50] Saslow MG (1967). Effects of components of displacement-step stimuli upon latency for saccadic eye movement. Journal Optical Social American.

[CR51] Sato A (2009). Both motor prediction and conceptual congruency between preview and action-effect contribute to explicit judgment of agency. Cognition.

[CR52] Schilbach, L., Wilms, M., Eickhoff, S. B., Romanzetti, S., Tepest, R., Bente, G., ... & Vogeley, K. (2010). Minds made for sharing: initiating joint attention recruits reward-related neurocircuitry. *Journal of Cognitive Neuroscience*, 22(12), 2702–2715. 10.1162/jocn.2009.21401 19929761

[CR53] Stephenson LJ, Edwards SG, Howard EE, Bayliss AP (2018). Eyes that bind us: Gaze leading induces an implicit sense of agency. Cognition.

[CR54] Suzuki K, Lush P, Seth AK, Roseboom W (2019). Intentional binding without intentional action. Psychological Science.

[CR55] Teufel C, Fletcher PC (2020). Forms of prediction in the nervous system. Nature Reviews Neuroscience.

[CR56] Ulloa JL, Vastano R, George N, Brass M (2019). The impact of eye contact on the sense of agency. Consciousness and Cognition.

[CR57] van der Wel RP (2015). Me and we: Metacognition and performance evaluation of joint actions. Cognition.

[CR58] van der Wel RP, Sebanz N, Knoblich G (2012). The sense of agency during skill learning in individuals and dyads. Consciousness and Cognition.

[CR59] Vesper, C., Van Der Wel, R. P., Knoblich, G., & Sebanz, N. (2011). Making oneself predictable: Reduced temporal variability facilitates joint action coordination. *Experimental Brain Research*, 211(3–4), 517–530. 10.1007/s00221-011-2706-z PMC3102185 21556820

[CR60] Vogeley K (2017). Two social brains: neural mechanisms of intersubjectivity. Philosophical Transactions of the Royal Society of B.

[CR61] Weller L, Schwarz KA, Kunde W, Pfister R (2020). Something from nothing: Agency for deliberate nonactions. Cognition.

[CR62] Wolpert DM, Doya K, Kawato M (2003). A unifying computational framework for motor control and social interaction. Phsilosophical Transactions of the Royal Society of London Series B: Biological Sciences.

[CR63] Yang Q, Bucci MP, Kapoula Z (2002). The latency of saccades, vergence, and combined eye movements in children and in adults. Investment Ophthalmologica Vision Science.

[CR64] Zakay D (2014). Psychological time as information: The case of boredom. Frontiers in Psychology.

